# Towards attaining universal health coverage in Kazakhstan: Challenges and important next steps

**DOI:** 10.34172/hpp.025.43529

**Published:** 2025-05-06

**Authors:** Usman Abubakar Haruna, Amos Abimbola Oladunni, Abdulafeez Katibi Abdulkadir, Abbas Bashir Umar, Shuaibu Saidu Musa, Elizabeth Oluwatoyin Afolabi, Joseph Almazan, Don Eliseo Lucero-Prisno III

**Affiliations:** ^1^Department of Clinical Pharmacy and Pharmacy Practice, Faculty of Pharmaceutical Sciences, Ahmadu Bello University, Zaria, Nigeria; ^2^Department of Biomedical Sciences, Nazarbayev University School of Medicine (NUSOM), Astana, Kazakhstan; ^3^Department of Pharmacy, Afe Babalola University Teaching Hospital, Ado-Ekiti, Ekiti State, Nigeria; ^4^Department of Public Health, Nazarbayev University School of Medicine (NUSOM), Astana, Kazakhstan; ^5^Nigerian Ports Authority Medical Centre, Lagos; ^6^School of Global Health, Faculty of Medicine, Chulalongkorn University, Bangkok, Thailand; ^7^Department of Clinical Pharmacy and Pharmacy Practice, Afe Babalola University, Ado-Ekiti, Ekiti State, Nigeria; ^8^Department of Global Health and Development, Faculty of Public Health and Policy, London School of Hygiene and Tropical Medicine, London, United Kingdom; ^9^Office for Research, Innovation and Extension Services, Southern Leyte State University, Sogod, Southern Leyte, Philippines; ^10^Center for University Research, University of Makati, Makati City, Philippines

**Keywords:** Health policy, Kazakhstan, Primary health care, Rural health services, Universal health coverage (UHC)

## Abstract

The Alma Ata Declaration (1978) positioned primary healthcare (PHC) as central to universal health coverage (UHC). Post-independence Kazakhstan struggles with a fragmented healthcare system marked by high mortality, underfunding, and workforce shortages. Despite initiatives like "Kazakhstan 2050" and "Salamatty Kazakhstan," challenges persist: economic instability, unregulated private healthcare, high out-of-pocket costs, and rural disparities. While Kazakhstan achieved a 76% UHC index, advancing PHC quality, expanding health financing, and prioritizing rural access remain critical to achieving equitable UHC.

## Introduction

 The demand for universal health coverage (UHC) began with the Alma Ata Declaration in 1978, when primary healthcare (PHC) facilities were identified as the strategy to achieve this goal. It emphasized the need for all governments, health and development workers, and the international community to take immediate action to protect and promote the health of all people. Kazakhstan, a central Asian country that gained independence from the former Soviet Union in 1991, has one of the world’s lowest population densities, with an estimated population of 19.2 million people.^1,2^ Prior to independence, Kazakhstan inherited a healthcare system that was severely underfunded and had several noticeable issues, including the dominance of inpatient care, inefficient service provision, and weak provider incentives.^2^ This period of difficult circumstances was characterized by a lack of adequate funding for healthcare and the emigration of skilled workers, resulting in high infant and adult mortality rates.^3^ The shortage of specialists and an unequal distribution of the healthcare workforce in the country have led to human resource challenges. Rural communities continue to be understaffed, whereas cities are much better staffed.^4,5^

 As part of the government’s efforts to realize the UHC goal, two comprehensive reform strategies were adopted from 2011 to 2015 as a component of the United National Health System (UNHS), considering two key indicators of healthcare excellence: quality and human development.^3^ The program aimed to increase life expectancy, reduce infant mortality rates, prevent HIV transmission, and combat tuberculosis and other socially dangerous diseases. Additionally, the government launched the “Kazakhstan 2050” strategy, which has led to the provision of quality and accessible medical services, the diagnosis and treatment of a wide range of diseases, the development of preventive medicine, the establishment of “Smart Medicine” services, distant prevention and treatment, and “e-Health.” This strategy ensured access to medical services for all children below the age of sixteen and legislates minimum living standards.^3^ So far, Kazakhstan has achieved a 76% index, making it closer to achieving the UHC goal.^6^

 Achieving UHC in Kazakhstan will require not only the provision of adequate PHCs but also strong health systems and solid financing structures. High out-of-pocket spending on healthcare services can significantly impact the population, especially the poor, pushing people further into poverty. While Kazakhstan has made a decent effort to reform the post-Soviet health system, key aspects of health indicators such as workforce, funding, health utilization, and health outcomes are still in dire need of enhancement.^4^ Only about 6000 facilities operate in rural areas, which do not fully address the problem of accessibility due to the low population density and sparse settlements.^3^ This narrative review aims to explore the challenges, efforts being made, and progress by Kazakhstan toward achieving UHC, thus providing recommendations to policymakers.

## Methods

 The aim of this study was to identify literature that outlines the challenges and potential solutions for enhancing healthcare access and quality in Kazakhstan, particularly in the context of UHC. A comprehensive online search was conducted using databases such as PubMed, Google Scholar, and Medscape. The search utilized keywords including “Universal Health Coverage,” “Primary Healthcare,” “Healthcare Reforms,” and “Kazakhstan.” The search was restricted to articles and reports published between January 2010 and January 2024, encompassing both peer-reviewed studies and reports from relevant organizations. Following a thorough screening of articles and abstracts, 17 articles were deemed relevant for inclusion in this narrative review. Additionally, 9 eligible references cited within these articles were incorporated to enrich the analysis.

## Results

###  Challenges to achieving UHC

 Robust healthcare systems, strong political commitment, disease preventive measures, and proper management of healthcare workers and resources are crucial to achieving UHC.^7^ Studies have stressed the significance of strong political commitment, increased government spending on health systems, economic growth, and a reduction in out-of-pocket costs in achieving universal health care in low- and middle-income countries (LMICs).^7^ Furthermore, community support and economic prosperity make healthcare accessible and play a vital role in achieving UHC. However, integration of those policies into the constitution is required for strong political support to prevail.^7^

 In most countries, particularly LMICs, basic healthcare is inadequately funded.^8^ Despite the declaration of Alma-Ata in 1978 and the implementation of UHC as a part of sustainable development goals (SDGs), UHC is still not achieved in Kazakhstan. Although various policies and targets have been set out to improve UHC, the country is faced with several challenges that continue to undermine its efforts towards achieving the UHC goal. These challenges include economic crisis, political instability, unregulated private health care, global pandemics, vertical disease-specific approaches, and an overabundance of specialized curative care.

 Funding for UHC in Kazakhstan is still inadequate, especially in rural areas, despite the proposal of President Nursultan Nazarbayev to increase the health budget in 2018. By the end of 2021, out-of-pocket expenses constituted 38% of total health expenditure, indicating poor government health spending. As of 2021, Kazakhstan still fell short of the 5% average of every country’s GDP recommended by the World Health Organization (WHO). Low government expenditure makes the out-of-pocket expenditure on getting basic healthcare high relative to financial aid provided by the government.^9^ This situation is further complicated by the emergence of the COVID-19 pandemic, stretching further the existing inadequate human and capital resources. In 2022, the majority of rural areas in north-eastern Kazakhstan have limited access to PHC needs.^3^ The system is being held back by a number of factors, including the high cost of insurance policies, the inability to receive money back if the insured event fails to occur, the lack of a voluntary healthcare insurance incentive healthcare package provided by the employer, and an increase in the actual value of a benefits package for the employer as a result of social taxes and deductions that are accumulated on insurance rates.^9^

###  Efforts being made toward achieving UHC

 Different policy implementations and interventions have been set to improve health promotion and disease prevention as part of UHC in Kazakhstan. These policies and future targets are aimed at improving UHC.^10,11^ Kazakhstan government has shown commitment towards attaining UHC goals by implementing targets such as reformation of the health sector, improvement of the financial budget, and acquisition of the state guarantee benefit package.^12,13^

 In addition, the government has ventured into promoting evidence-based medicine, introducing newer and better clinical practice guidelines, and also improving facility quality levels. However, some key areas such as health financing, healthcare utilization, and health outcomes remain underdeveloped in some regions, drawing back years of progress towards achieving UHC. Some successful efforts and policies include the building of new hospitals and clinics, implementation of a unified national health system, provision of free basic health services, implementation of a state health care development program (Salamatty Kazakhstan), establishment of innovative health facilities, increased health financing, and introduction of transport medicine aimed at providing health services for remote areas in the country.

 The government also implemented a partial fund management system to improve the prevention of maternity and infant mortality, quality care of patients, early diagnosis of socially significant diseases such as cancer, HIV, HCV, and TB, and prevention of cardiovascular disease complications. However, the successful implementation of this initiative was undermined by insufficient funding and underdeveloped primary health care.^9^ In addition, Kazakhstan implemented special surveillance systems, conducted confidential inquiries on maternal death data, and also introduced National Diagnosis Related Groups (DRG). The implementation of this program has helped to produce more accurate data systems, revise clinical guidelines, improve financial budgets for the health sectors, and standardize strategies such as fee exemptions, benefit packages, and worker’s insurance in order to improve the healthcare system and achieve UHC.^7^

## Progress by Kazakhstan toward achieving UHC and discussion

 Kazakhstan’s health care system consists of a network of primary, secondary, and tertiary care facilities. The Ministry of Health, which represents the public sector, owns and operates the majority of healthcare institutions. In the public sector, health insurance is currently predominantly provided by the government. According to the WHO, in 2022, the country’s healthcare system ranked 64th in overall performance and 135th in terms of overall health among 191 member nations.^7^

 Following the collapse of the Soviet Union in the early 1990s, Kazakhstan was one of five Central Asian countries that inherited the Centralized Soviet Union health system.^14,15,16^ This system was characterized by centrally controlled programs with limited patients’ choices when seeking health care.^17^ After gaining sovereignty, several health reforms were implemented that led to changes in the country’s health system. From 1991 to 1996, the country’s health system was managed by the Public Health Ministry. In 1996, the government introduced the Insurance Reform, which was characterized by mandatory and voluntary insurance under the Compulsory Medical Insurance Fund. This program failed but was a positive indicator of financial independence. In 1999, the Public Health Ministry was restructured and modified to become the Ministry of Public Health, Education, and Sport of the Republic of Kazakhstan under the Development Strategy of the Republic of Kazakhstan with the long-term goal of preserving health and ensuring the well-being of the population by 2030. In addition, the Health of the Nation initiative was adopted to improve the health status of the population of Kazakhstan through the development and implementation of short-, medium-, and long-term plans to foster universal access to healthcare among the population.^3^

 The implementation of the Health of the Nation-State program and the concept of further development of health care in the Republic of Kazakhstan were the primary focus of the healthcare sector’s activity during this time.^18^ The program’s goals are to improve Kazakhstan’s overall healthcare system by developing primary medical care, providing a modern level of medical care, addressing the issue of unhealthy lifestyles, realizing the constitutional right to health care, and improving the quality and service provided by health practitioners to the general public.^3^ In 2022, the protection of the population’s health was nominated to the list of priority goals of national importance, which marked a turning point in the medical field.

 After the first phase of reform, Kazakhstan adopted two health reform programs, namely the National Program for Reforming and Developing Healthcare in the Republic of Kazakhstan from 2005–2010 and the National Program of Healthcare Development, also known as “Salamatty Kazakhstan,” from 2011–2015.^19^ The aim of these programs is to create an effective system of providing medical care based on the principles of joint liability for health care between the government and its citizens, the development of public healthcare as a priority directed to improve the main health indicators of the population, and improving the health of the people of Kazakhstan by ensuring stable social-demographic development and forming a competitive healthcare system.^20^ These two programs recorded success to some extent, but there were still challenges like inequalities in healthcare financing at the regional level and the use of healthcare services and healthcare outcomes.^19^ As a result, the Ministry of Health and Social Development Kazakhstan established the National Program of Healthcare Development from 2016 to 2019.^14^ The goal of this program is to promote population health and the sustainable social and economic development of the people of Kazakhstan. This program was said to be a logical extension of earlier government reform initiatives as well as the growth of public health for the years 2005–2010 and “Salamatty Kazakhstan’’ for the years 2011–2015.^18^

 Despite the high level of success recorded by this program, policy-making and funding remain largely concentrated within Kazakhstan’s ministry of health, despite many waves of reform.^14^ According to the human rights measurement initiatives in 2021, Kazakhstan is performing poorly, achieving 76.8 percent of what it should for the right to health based on its income.^21^ Albeit the country is measured at 96.1% and 80.9% of what is expected based on its existing income when it comes to the right to health for children and adult population respectively.^14,21^ However, the tough epidemiological condition of COVID-19 across the globe has reshaped the healthcare system in Kazakhstan. In response to the unprecedented effect of the pandemic, the government, through the ministry of health, took an updated measure to improve the overall system of the country by strengthening the healthcare systems, including technical and human resources.

 The format of medical care was updated at this time.^19^ The provision of advisory services via the National Telemedicine Network, which is connected to 259 health institutions, began to take off. Patients seeking treatment in regional hospitals are often consulted by specialists from nearby clinics. In order to assure the availability of primary health care in 2020, 44 outpatient care facilities, including 30 in rural locations, were developed and opened. The average compensation for doctors increased by 30% in 2020, while the average income for nurses increased by 20%. Measures were also taken in 2021 to upgrade the health infrastructure as well as the material and technical support provided to medical organizations in the regions.^14,19^ These steps are intended to improve the quality and accessibility of medical care.

## Discussion

###  Main findings 

 Primary health care includes community engagement, multi-sectoral policies and activities, and the coordinated implementation of high-quality primary care and public medical services. However, no country can attain UHC without prioritizing PHC.^8^ Study has shown that rural settlements in Kazakhstan have less access to healthcare services due to a lower standard of living compared to urban settlements, indicating that some healthcare services will be unaffordable, resulting in health inequalities.^13^ Also, Turgambayeva et al conducted a study to assess the quality of life of people living in rural settlements of Kazakhstan during the time of COVID-19; the authors stated that the quality of life for people living in rural areas is still relatively low because of a lack of employment opportunities, middle wages, and the issue with the water supply.^22^ Fully functioning PHC facilities should be built and equipped around all rural settlements in Kazakhstan. The facilities should also be manned by healthcare professionals. Healthcare personnel should be deployed to every area of the country, including PHC facilities in rural settlements, so that the people of those areas could have easy and equitable access to medical care services. This will ensure access to healthcare and reduce the overall burden on secondary and tertiary institutions.

###  Health system and pharmaceutical sector reforms 

 The primary aim of UHC is to provide high-quality and equitable health care for everyone, regardless of their social or economic status. Hence, efforts should be made to enrol every citizen of Kazakhstan in the health insurance scheme, where funds could be pooled to make medical services and pharmaceutical products affordable and accessible in the country. There is also a need to educate the citizens of Kazakhstan, especially those in rural settlements, on the advantages and benefits of insurance coverage and the risk associated with being uninsured. Additionally, the government of Kazakhstan should liaise with pharmaceutical companies by signing a memorandum of understanding such that affordable medicines and medical consumables will always be available in every health facility across the country. Additionally, the government needs to come up with policies that will regulate private healthcare facilities in Kazakhstan. The government should collaborate with the private sector to make healthcare accessible and affordable as per the UHC goals. This collaboration will be to ensure that out-of-pocket spending expenditures are reduced in the patients visiting private clinics, and the patients visiting private clinics should also be insured.

 Despite the progress made in Kazakhstan’s healthcare reforms, significant knowledge gaps persist, particularly regarding the effectiveness of these reforms in different geographical contexts. The disparities in healthcare access and quality between urban and rural areas highlight the need for targeted research to better understand the unique challenges faced by rural populations.^23^ Additionally, the impact of global health crises, such as the COVID-19 pandemic, on healthcare delivery and outcomes in Kazakhstan requires further investigation to inform future policy decisions.^24^ Kazakhstan’s healthcare reforms, while ambitious, have not yet achieved the equitable health outcomes seen in some neighboring Central Asian countries. For instance, Kyrgyzstan’s focus on community-based health initiatives has resulted in better access to healthcare services in rural areas compared to Kazakhstan.^4,25^ Globally, Kazakhstan’s high levels of out-of-pocket healthcare expenditure are consistent with trends observed in other LMICs, where inadequate government health spending exacerbates health inequalities.^25^

###  Increased government spending and reduction of out-of-pocket expenditures

 The government of Kazakhstan needs to increase budgetary allocation for health and financial commitment, especially in the rural settlements where they are lacking even basic healthcare facilities. Special groups such as pregnant women, children, low-income earners, and old people should be exempt from paying for medical care services. Out-of-pocket expenditure reduction can be achieved through the procurement of generic drugs, which are cheaper compared to branded drugs. Enlightening Kazakhstani citizens citizens on the importance of preventive measures for various types of health conditions will also result in a reduction in out-of-pocket spending.

###  Leveraging technology to improve healthcare access

 Healthcare facilities are sometimes overstretched with the influx of patients from varying walks of life, each with varying medical emergencies. Mobile communication technologies can be used to increase access to healthcare, especially in medical emergencies. These services could be explored through phone calls, text message video calls, or by using mobile phone applications. By leveraging such technologies, people could access medical emergencies on time, and they would always be within reach, especially for people in rural settlements. This directly translates to fewer patients visiting emergency departments and the availability of medical services even in the comfort of their own homes.

## Study limitations

 This narrative review’s findings are subject to several limitations. The reliance on secondary data, primarily published articles and reports, introduces potential biases due to selection bias and publication bias, hindering a fully representative understanding of UHC in Kazakhstan. The narrative review methodology itself prevents establishing causal relationships between policies and outcomes, as correlations may be influenced by confounding factors. The focus on policy-level analysis overlooks grassroots perspectives, limiting the understanding of lived experiences accessing healthcare, particularly in rural areas. The rapidly changing healthcare landscape, including the impact of the COVID-19 pandemic and time lags in data, challenges the generalizability and timeliness of the findings. Finally, the possibility of missing relevant studies due to the limitations of relying on existing literature necessitates future research employing more robust methodologies like systematic reviews or meta-analyses for a more comprehensive assessment of UHC progress in Kazakhstan.

## Conclusion

 The Kazakhstan government has made significant efforts towards achieving UHC even in the face of daunting health system challenges. However, there is a need to intensify efforts to ensure provision of basic medical services to all who need them. There is a need to review the healthcare budget allocation in order to achieve the WHO recommended benchmark. Also, the State Guaranteed Health Benefits package and related health insurance must be expanded to cover more procedures, drugs, and consumables to reduce out-of-pocket spending and ensure universal access to healthcare.

## Competing Interests

 The authors declare that they have no competing interests.

## Ethical Approval

 This study used data freely accessible and downloadable in the public domain. Therefore, institutional review board approval was not required.
